# Microwave Assisted Synthesis, Pharmacological Activities, and Molecular Docking Studies of Ethyl 2-[2-Substituted-4-(Thiophenyl) Thiazolyl] Acetates

**Published:** 2017

**Authors:** Mahesh Veerabhadra Attimarad, Mohammed Abdou Khedr, Bandar Essa Aldhubiab

**Affiliations:** *College of Clinical Pharmacy, King Faisal University, Al-Ahsa, KSA.*

**Keywords:** Analgesic, Anti-inflammatory, Antioxidant, Molecular modeling, Synthesis, Thiazole acetates

## Abstract

A series of ethyl 2-[2-arylamino-4-(thiophen-2-yl) thiazol-5-yl] acetates (5a-5k) and ethyl 2-[2-(arylhydrazino)-4-(thiophen-2-yl) thiazol-5-yl] acetates (9a-9g) were synthesized and screened for their anti-inflammatory, analgesic, and antioxidant activities. *In-vivo *test results showed that the compounds with halogen substitution (5c, 5g, 5h, 5i and 5j) at the para position on the 2-aryl amino group exhibited good anti-inflammatory and analgesic activities, similar to that of indomethacin and aspirin, respectively. The ethyl 2-[2-(arylhydrazino)-4-(thiophen-2-yl) thiazol-5-yl] acetates (9a-9g) showed better anti-oxidant activity than compounds 5a-5k, comparable to ascorbic acid. However, these compounds showed moderate to weak anti-inflammatory and analgesic activities. Further, a molecular docking study was performed to predict the possible binding modes on cyclooxygenase-1 (COX-1) and COX-2 for the tested compounds. Good correlation was observed between the anti-inflammatory activity of the compounds and the results of the binding modes in COX-2.

## Introduction

Non steroidal anti-inflammatory drugs (NSAIDs), which generally have anti-inflammatory, analgesic, and antipyretic properties, are commonly employed for the management of fever, pain, and inflammation of arthritis and rheumatism diseases (1-3). NSAIDs mainly act by inhibiting the cyclooxygenase (COX) enzyme, which mediates the initial step of converting arachidinoic acid into prostaglandins (4), a precursor for the synthesis of other prostaglandins and thromboxanes (5). It is well established that COX exists in two isoforms, COX-1 and COX-2 (6). The constitutive COX-1 isoform is found in normal tissues and is required for many regular physiological functions related to the excretory system and digestive system (7). Conversely, the COX-2 isoenzyme is induced by noxious stimulus and is responsible for the formation of prostaglandins, a pain and inflammation mediator (8). Selective and non-selective NSAIDs are available on the market for the treatment of various inflammatory diseases. The COXIB class of NSAIDs consists of selective COX-2 inhibiters (9-10), whereas classic carboxylic acid derivatives are non-selective NSAIDs that are still used for the management of inflammatory diseases. Side effects, such as gastric ulceration and suppression of renal function, associated with non-selective NSAIDs are due to the inhibition of COX-1 (11-13). Hence, the recent trend of the development of NSAIDs focuses on the development of selective COX-2 inhibitors (14-16). However, the long-term use of some selective COX-2 inhibitors have showed a number of unwanted side effects, such as ulceration in sensitive patents, slow healing of stomach ulcers, kidney, and cardiovascular toxicity (17-19). Hence, rofecoxib and some of the selective COX-2 inhibitors have been withdrawn from the market due to their adverse side effects (20-21). Since this time, all selective COX-2 inhibitors have been under examination. Further, all selective and non-selective NSAIDs are classified under one group by the United States Food and Drug Administration and have been accompanied by the same caution regarding cardiovascular, renal, and gastrointestinal side effects (22). Therefore, the search for more effective compounds with fewer risks continues.

Many thiazole derivatives with various pharmacodynamic nuclei are reported to possess diverse pharmacological activities, such as anti-inflammatory, anticancer, antibacterial, antifungal, and antioxidant activities (23-28). Frankline *et al*. (29) reported the structure activity relationship and anti-inflammatory activity of a series of substituted thiazole derivatives; it is evident from the data that the 2-substituted amino thiazolyl moiety is required for the anti-inflammatory activity. Recently, 2-amino substituted thiazole derivative studies have shown good anti-inflammatory activity (29-30). During the past three decades, microwave assisted organic synthesis has been extensively used due to its simplicity, reduced reaction time, and increased yield. Driven by these citations and in the continuous search for potent therapeutic molecules, we planned to synthesize title compounds with the 2-substituted amino thiazole with carboxylic acid (as ester) moieties by microwave irradiation, and we have screened them for their anti-inflammatory, analgesic, and antioxidant activities.

The molecular modeling approach has been widely used for the prediction and interpretation of the pharmacological activities of active compounds. Molecular docking is one of these approaches and can be used for the prediction of the orientation of different conformations inside the active site to identify the best mode of binding. Good correlation between computational and biological activities can be explained depending on the computed binding affinities. Hence, molecular docking was also included as one of our aims to predict the binding modes for all the tested compounds and also to interpret the high activity of the top active compounds when compared to indomethacin.

## Experimental

*Chemistry*

The reactions were carried out in microwave test tube containing magnetic stirrer fitted with rubber cap in the CEM Discovery microwave system. Melting points were recorded in open capillaries and were uncorrected. The infrared (IR) spectra were scanned using a Shimadzu FT IR spectrophotometer in KBr pellets. ^1^H NMR was obtained using JEOL GSX-400 FT NMR 400 MHz in CDCl_3_ solvent using Tetra methyl Silane as an internal reference. Mass spectra were recorded by a JEOL-JMS-300 spectrometer at 70 eV. Elemental analysis was performed for the selected compounds and values were found to be very close to theoretical values. TLC on silica gel plates was used to check the progress of the reaction and purity of compounds.

**Table 1:** Physical Data of compounds 5a-5k and 9a-9g.

**Compd No**	**X**	**Mol. Formula**	**Mol. Weight**	**Irradiation Time**	**Yield %**	**m.p. (** ^O^ **C)**
**5a**	2-Cl	C_17_H_15_N_2_O_2_S_2_Cl	378.90	1 min	92	90-92
**5b **	3-Cl	C_17_H_15_N_2_O_2_S_2_Cl	378.90	1 min	89	118-120
**5c **	4-Cl	C_17_H_15_N_2_O_2_S_2_Cl	378.90	1 min	84	146-147
**5d **	2-CH_3_	C_18_H_18_N_2_O_2_S_2_	358.48	1 min	93	107-108
**5e **	3-CH_3_	C_18_H_18_N_2_O_2_S_2_	358.48	1 min	95	122-123
**5f **	4-CH_3_	C_18_H_18_N_2_O_2_S_2_	358.48	1 min	86	174-176
**5g **	4-Br	C_17_H_15_N_2_O_2_S_2_Br	423.44	1 min	73	155-156
**5h **	4-F	C_17_H_15_N_2_O_2_S_2_F	362.44	1 min	83	135-136
**5i **	2,4-Cl_2_	C_17_H_14_N_2_O_2_S_2_Cl_2_	413.35	1.5 min	90	84-85
**5j **	3-Cl-4-F	C_17_H_14_N_2_O_2_S_2_ClF	396.89	1.5 min	79	115-116
**5k **	4-OCH_3_	C_18_H_18_N_2_O_3_S_2_	374.48	1 min	90	153-154
**9a**	H	C_18_H_17_O_2_N_3_S_2_	371.47	40 sec	91	163-164
**9b**	2-OH	C_18_H_17_O_3_N_3_S_2_	387.47	40 sec	89	152-153
**9c**	2-Cl	C_18_H_16_O_2_N_3_S_2_Cl	405.91	40 sec	92	149-150
**9d**	4-Cl	C_18_H_16_O_2_N_3_S_2_Cl	405.91	40 sec	79	171-172
**9e**	4-NMe_2_	C_20_H_22_O_2_N_4_S_2_	414.54	40 sec	85	184-185
**9f**	4-OMe	C_19_H_19_O_3_N_3_S_2_	401.50	40 sec	90	146-147
**9g**	4-OH-3-OMe	C_19_H_19_O_4_N_3_S_2_	417.49	40 sec	83	162-163

Compounds 5a-k, 9a-9c were crystallized from absolute ethanol. 9d-9g were crystallized from Ethanol+Acetone. Melting points were determined in an open capillary and are uncorrected. Reactions are monitored on silica gel G TLC plates using solvent system ethyl acetate: n-hexane (3: 7

**Table 2:** Pharmacological activities of compounds 5a-5k and 9a-9g.

**Compd.**	**Mean paw volume ± SEM**	**% Anti-Inflammatory Activity**	**Writhing ± SEM**	**% Analgesic Activity**	**Anti-oxidant Activity ± SD (IC**_50_ ** in μg/mL)** *
**5a**	0.22 ± 0.060	66.6	22.5 ± 2.54	52.54	160±2.78
**5b**	0.21 ± 0.074	68.1	24.3 ± 4.37	49.35	190±3.16
**5c**	0.18 ± 0.069	72.7	21.8 ± 2.39	54.50	120±2.95
**5d**	0.28 ± 0.056	54.9	32.7 ± 3.57	31.8	200±1.74
**5e**	0.20 ± 0.079	69.6	24.6 ± 4.02	48.70	255±2.45
**5f**	0.24 ± 0.074	63.4	26.3 ± 3.82	45.20	140±1.98
**5g**	0.14 ± 0.068	78.8	20.6 ± 2.65	57.00	130±1.62
**5h**	0.15 ± 0.070	77.2	21.10 ± 3.54	56.04	95±1.04
**5i**	0.18 ±0.075	72.7	29.3 ± 2.54	39.10	90±1.67
**5j**	0.17 ±0.047	74.3	21.60 ± 3.44	55.00	50±1.50
**5k**	0.29 ± 0.072	50.1	25.9 ± 2.24	46.00	145±2.83
**9a**	0.31 ± 0.076	53.3	28.1 ± 2.69	41.50	8.25±0.29
**9b**	0.35 ± 0.072	46.9	30.7 ± 3.00	36.00	11.75±0.31
**9c**	0.30 ± 0.079	54.5	29.3 ± 2.54	39.10	10.50±0.36
**9d**	0.32 ± 0.082	51.5	31.6 ± 3.36	34.10	NT
**9e**	0.40 ± 0.064	39.3	35.5 ± 4.51	26.00	NT
**9f**	0.48 ± 0.077	27.3	23.3 ± 4.03	51.40	NT
**9g**	0.42 ± 0.083	36.36	26.0 ± 3.38	45.70	10.32±0.21
Indomethacin	0.12 ± 0.051	82.6	-	-	
Aspirin	-	-	22.00 ± 2.19	54.1	
Ascorbic acid	-	-	-	-	3.3±0.07

NT: Not Tested.

* value are average of three experiments ± standard deviation.

**Table 3:** Docking Results, lipophilic contribution, clash penalties of all compounds on COX-2 with Leadit 2.1.2 software.

**Compd.**	**Docking Score kcal/mol**	**Lipo Score**	**Clash Penalty**	**Interacted moiety ** **&** **Involved Residues**
5a	-14.96	-16.77	8.35	The ‘’O’’ of acetate moiety interacted with ‘’HN-‘’ of His 90 ‘’S’’ atom of thiophene ring with ‘’-OH’’ of Tyr 355
5b	-13.99	-15.48	8.64	The ‘’S’’ atom of thiophene with both –NH of Arg 120 and –OH of Tyr 355
5c	-20.02	-18.61	6.49	The ‘’S’’ atom of thiophene with both –NH of Arg 120 and –OH of Tyr 355
5d	-14.84	-16.35	10.63	The ‘’O’’ atom of acetate with –OH of Tyr 355
5e	-16.81	-15.19	9.49	The 2-arylamino –NH with –C=O of Met 522. The ‘’S’’ atom of thiophene with both –NH (of Arg 120) and –OH of Tyr 355
5f	-15.65	-15.32	9.60	The C=O of acetate with both –NH of Arg 120 and –OH of Tyr 355
5g	-21.31	-18.62	5.01	The 2-arylamino –NH with ‘’O’’ of Ser 530 The ‘’S’’ atom of thiophene with both –NH of Arg 120 and –OH of Tyr 355
5h	-21.52	-19.86	5.21	The 2-arylamino –NH with ‘’O’’ of Ser 350 The ‘’S’’ atom of thiophene with both –NH of Arg 120 and –OH of Tyr 355
5i	-18.74	-17.38	7.21	The ‘’S’’ atom of thiophene with two –NH of guanidine moiety from Arg 120
5j	-16.94	-18.80	6.67	The ‘’S’’ atom of thiophene with both –NH of Arg 120 and –OH of Tyr 355 The 2-arylamino –NH with the ‘’O’’ atom of Ser 530
5k	-14.31	-15.90	9.81	The ‘’S’’ atom of thiophene with both –NH of Arg 120 and –OH of Tyr 355
9a	-14.42	-14.93	12.55	The benzylidene C=N with both –NH of Arg 120 and –OH of Tyr 355
9b	-14.86	-14.55	11.26	The benzylidene C=N with–OH of Tyr 355 The ‘’O’’ of ortho –OH with –OH of Tyr 355 The ‘’O’’ of acetate with –OH of Ser 530
9c	-12.10	-12.37	13.66	The benzylidene C=N with–OH of Tyr 355 The ‘’O’’ of acetate with –OH of Ser 530
9d	-5.45	-13.74	14.31	The benzylidene C=N with–OH of Tyr 355
9e	-12.37	-10.32	14.08	The benzylidene C=N with–NH of Arg 120 The C=N of thiazole ring with –OH of Tyr 355
9f	-8.96	-13.49	12.48	The C=O of acetate with both –OH (Tyr 355) and –NH (Arg 120).
9g	-5.79	-12.48	14.60	The para-OH with C=O group of Gln 192
Indomethacin	-27.25	-13.64	7.31	COO interacts with both –NH2 (Arg 120) and –OH (Tyr 355).

**Table 4:** The docking scores, lipophilic contribution, clash penalties of the studied compounds on COX -1 with Leadit 2.1.2 software

**Compd.**	**Docking Score kcal/mol**	**Lipo Score**	**Clash Penalty**	**Interacted moiety** **&** **Involved Residues**
5c	-13.10	-14.01	8.35	The 2-arylamino-NH with Met 522
5g	-15.00	-14.16	9.6	The ‘’S’’ atom of thiophene with –NH of Arg 120
5h	-13.87	-15.97	8.84	The ‘’S’’ atom of thiophene with –NH of Arg 120 The 2-arylamino -NH with oxygen atom of Ser 530
5i	-8.98	-14.63	12.17	The ‘’S’’ atom of thiophene with –NH of Arg 120 The C=N of thiazole ring with –NH of Arg 120
5j	-11.95	-14.21	9.01	The 2-arylamino -NH with Met 522
Indomethacin	-22.39	-13.23	7.22	COO interacts with both –NH and NH_2_ Arg 120

**Scheme 1:**
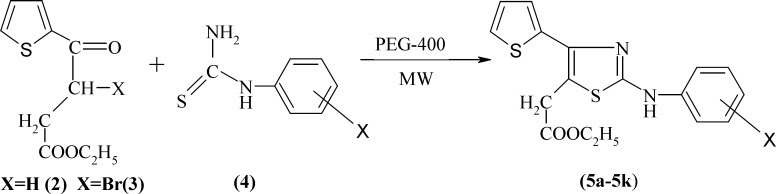
The synthetic pathway of compounds 5a-5k

**Scheme 2:**
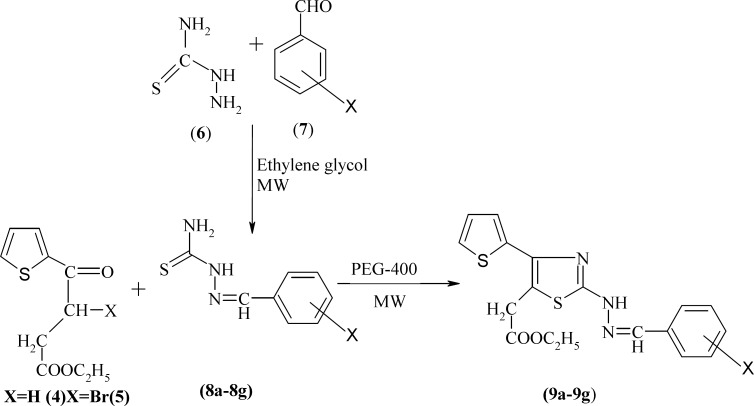
The synthetic pathway of compounds 9a-9g

**Scheme 3:**
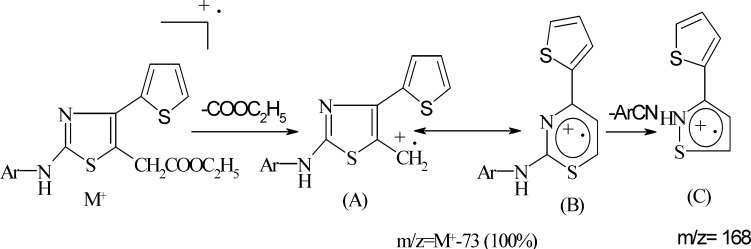
Probable mass fragmentation of componud 5a-5k

**Scheme 4:**
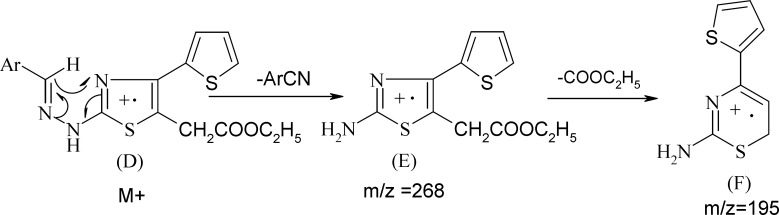
Propbable mass fragmantation of compound 9a-9g

**Figure 1:**
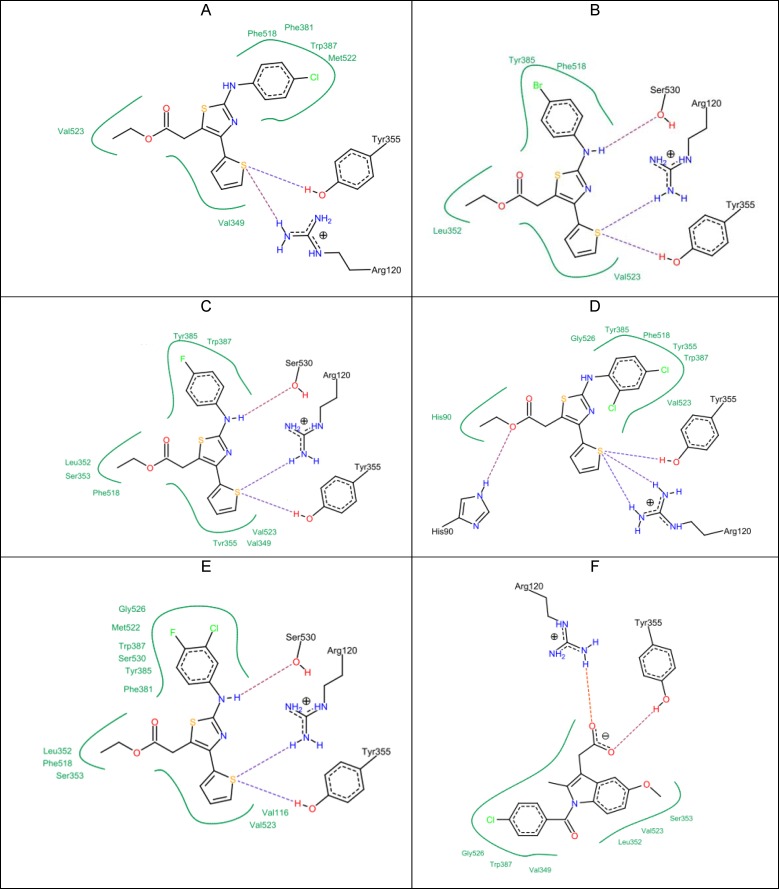
Docking results of the top active compounds against COX-2 enzyme showing the best binding modes for each compound A) compound 5c. B) compound 5g. C) compound 5h. D) compound 5i. E) compound 5j. F) Indomethacin

**Figure 2:**
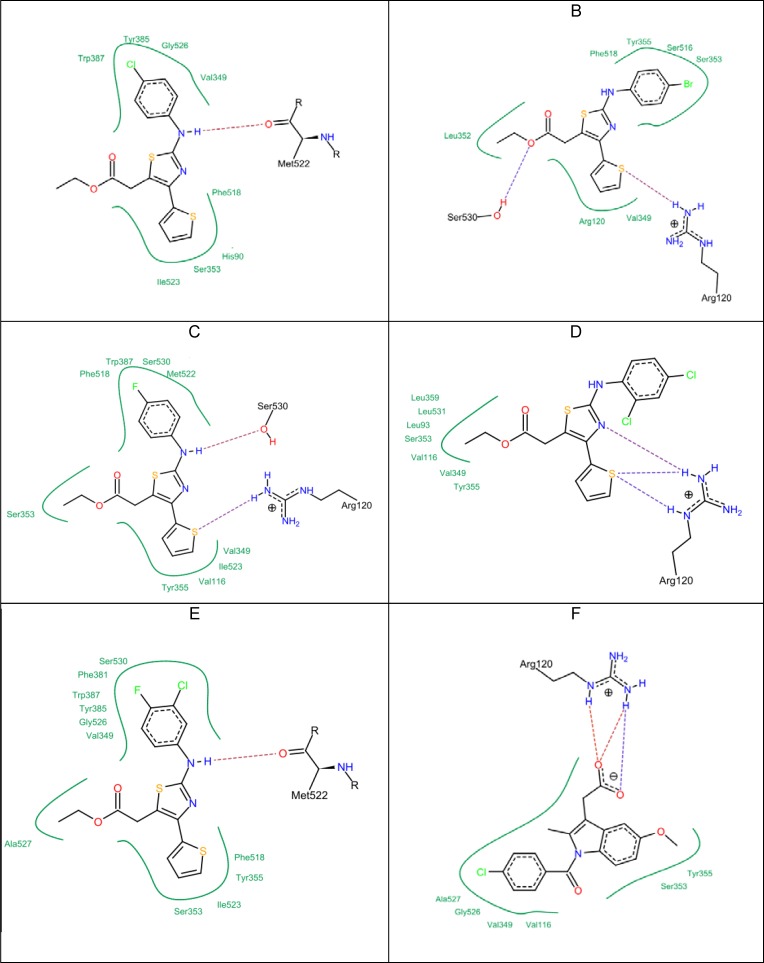
Docking results of the top active compounds against COX-1 enzyme showing the best binding modes for each compound A) compound 5c. B) compound 5g. C) compound 5h. D) compound 5i. E) compound 5j. F) Indomethacin

**Figure 3:**
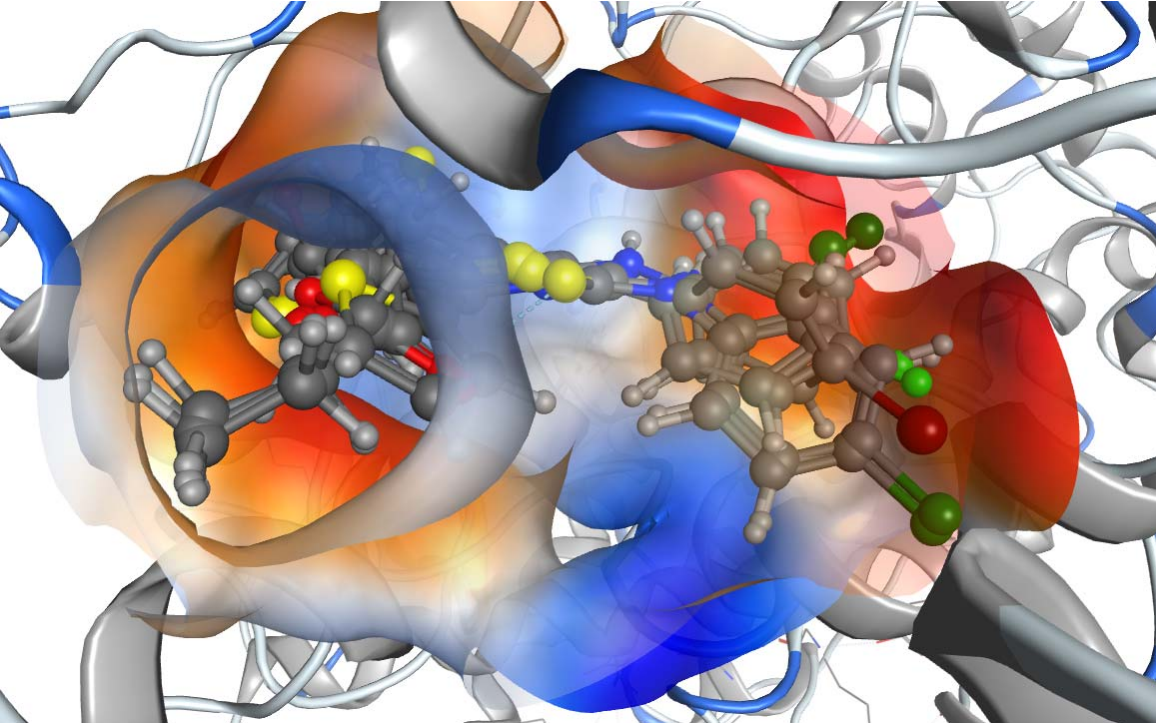
The lipophilicity map of the binding site of COX-2 enzyme with compounds 5a, 5g, 5h, 5i and 5j. The more lipophilic parts (in red), hydrophilic parts (in blue) and moderate lipophilic parts (in orange

*Synthesis of ethyl 3(2-thenoyl) propionate (2)*

A solution of 3-(2-thienoyl) propionic acid (0.15 moL) in 10 mL of absolute ethanol and 0.5 mL of concentrated sulfuric acid was irradiated with MW at 80 °C for 15 min. The hot irradiated solution was cooled and then transferred in to 500 mL ice-cold water. The ester separated like oil and was extracted in diethyl ether. Initially, the ether layer was washed with water and finally with a saturated solution of sodium bicarbonate. The ether layer was dried over anhydrous sodium sulfate and evaporated in a vacuum to obtain ethyl 3-(thiophen-2-yl) propionate 2 as an oil at 89% yield.

*Synthesis of ethyl 3-bromo-3-(thiophen-2-yl) propionate (3)*

Bromine (0.011 mol) was added drop-wise with constant stirring to a solution of ethyl 3-(2-thienoyl) propionate 2 (0.01 mol) in warm chloroform (20 mL). After adding all of the bromine, the reaction mixture was stirred for another 2 h and then the solution was washed with water to remove hydrogen bromide. The chloroform was distilled after drying the reaction mixture over anhydrous sodium sulfate to get bromoester 3 at 85% as thick oil, which was employed immediately for the subsequent reactions. 

*General synthesis of ethyl -2-[2-(substituted phenylamino)-4-(thiophen-2-yl) thiazol-5-yl] acetates (5a-5k)*

A mixture of bromo ester 3 (5 mmol) and substituted phenylthiourea 4** (**5 mmol) in polyethylene glycol (PEG)-400 (15 mL) was exposed microwave at 100 °C (power 100 W) for 60 sec. Upon completion of the irradiation, the cooled reaction solution was triturated with sodium carbonate solution. The reaction mixture was kept a side for 15 min to separate the product completely, which was then filtered, washed with water, dried, and crystallized to form aqueous ethanol to afford pure thiazole acetates 5a-5k; the physical constants are recorded in Table 1.

*Ethyl2-[2-(2-chlorophenylamino)-4-(thiophen-2-yl) thiazol-5-yl] acetate (5a):* IR (KBr pellets) ν, cm^-1^: 3355 (NH str), 2981 (C-H str), 1730 (ester C=O str), 1589, 1529, 1463 (C= and, Aromatic C=C str), 854 (Aromatic Cl str). ^1^H NMR (400MHz, CDCl_3_, δ ppm): 1.25, (t, 3H, CH_2_CH_3, _J = 7.0 Hz)_, _3.7 (s, 2H, CH_2_), 4.13 (q, 2H, CH_2_CH_3,_ J= 7.05 Hz), 7.0-7.8 (m, 8H, Ar-H and NH). ^13^C NMR (400MHz, DMSO-d_6_ δ ppm) δ(DEPT):161.712 (C_2,_ Q); 140.995 (C_4_, Q); 115.416 (C_5_, Q); 31.873 (C_6,_ CH_2_); 169.679 ( C_7_, C=O); 60.823 (C_8_, CH_2_); 13.997 (C_9_, CH_3_); 138.740 (C_1’_, Q); 112.655 (C_2’_, Q); 129.623 (C_3’­_ , CH); 146.955 (C_4’_, CH); 127.776 (C_5’_, CH); 121.314 (C_6’_, CH); 137.734 (Cb, CH); 127.924 (Cc, CH); 126.118 (Cd, CH); 124.987 ( Ce, CH). ESI-MS (m/z) 379/377 (M^+^+2/M^+^), 336/334 (100%, M^+^ - COOC_2_H_5_)

*Ethyl2-[2-(3-chlorophenylamino)-4-(thiophen-2-yl) thiazol-5-yl] acetate 5b:* IR (KBr pellets) ν, cm^-1^: 3211 (NH str), 2950 (C-H str), 1735 (C = O str), 1579, 1527, 1502, 1471 (C=N, Aromatic C = C str) 829 (Ar-Cl). ^1^HNMR (400MHz, CDCl_3_, δ ppm): 1.25 (t, 3H, CH_2_CH_3,_ J=7.0 Hz), 3.7 (s, 2H, CH_2_), 4.13 (q, 2H, CH_2_CH_3,_ J=7.05 Hz), 7.0-7.8 (m, 6H, aromatic), 9.5-9.7 (hump, 1H, NH). ^13^C NMR (400MHz, DMSO-d_6_) δ ppm (DEPT):160.668 (C_2,_ Q); 141.174 (C_4_, Q); 118.884 (C_5_, Q); 32.182 (C_6,_ CH_2_); 170.132 ( C_7_, C = O); 60.881 (C_8_, CH_2_); 14.170 (C_9_, CH_3_); 142.489 (C_1’_, Q); 116.197 (C_2’ _, CH); 133.351 (C_3’­_ ,Q); 120.605 (C_4’_, CH); 130.495 (C_5’_, CH); 115.275 (C_6’_, CH); 138.075 (Cb, CH); 127.893 (Cc, CH); 125.908 (Cd, CH); 124.781 ( Ce, CH).

*Ethyl2-[2-(o-toludino)-4-(thiophen-2-yl) thiazol-5-yl] acetate 5d:* IR(KBr pellets) ν, cm^-1^: 3166 (NH str), 2929 (C-H str), 1733 (C = O str), 1558, 1458 (C = N, Aromatic C=C str).^1^H NMR (400MHz, CDCl_3_, δ ppm): 1.28, (t, 3H, CH_2_CH_3,_*J* = 7.1 Hz,)_, _2.35 (s, 3H, CH_3_) 3.85 (s, 2H,CH_2_), 4.2 (q, 2H, CH_2_CH_3,_*J*= 7.1 Hz), 6.9-7.6 (m, 8H, Ar-H and NH).

*Ethyl2-[2-(m-toludino)-4-(thiophen-2-yl) thiazol-5-yl] acetate 5e:* IR (KBr pellets) ν, cm^-1^: 3166 (NH str), 2922 (C-H str), 1732 (C = O str), 1593, 1546, 1471 (C=N, Aromatic C=C str). ^1^H NMR (400 MHz, CDCl_3_, δ ppm):1.20, (t, 3H, CH_2_CH_3, J_ = 7.12 Hz,)_, _2.3 (s, 3H, CH_3_) 3.73 (s, 2H, CH2), 4.2 (q, 2H, CH_2_CH_3,_* J*= 7.11 Hz), 6.87 -7.50 (m, 7H, Ar-H), 7.81 (br, 1H, NH). ESI-MS (m/z). 358 (M^+^), 285 (100%, M^+^ - COOC_2_H_5_). Anal. Calcd. %:(C_18_H_18_N_2_O_2_S_2_): C, 60.31; H, 5.06; N, 8.93; S, 17.89.Found (%): C, 60.38; H, 5.12; N, 8.87; S, 17.95.

*Ethyl2-[2-(p-toludino)-4-(thiophen-2-yl) thiazol-5-yl] acetate 5f:* IR (KBr pellets) ν, cm^-1^: 3189 (NH str), 2918 (C-H str), 1727 (C=O str), 1603, 1559, 1483 (C=N, Aromatic C=C str). H NMR (400MHz, CDCl_3_, δ ppm): 1.26, (t, 3H, CH_2_CH_3,_* J *= 7.13 Hz)_, _2.32 (s, 3H, CH_3_) 3.78 (s, 2H, CH_2_), 4.15 (q, 2H, CH_2_CH_3 _*J*= 7.14 Hz), 7.07 -7.56 (m, 7H, Ar-H), 7.83 (br, 1H, NH). 

*Ethyl2-[2-(4-bromophenylamino)-4-(thiophen-2-yl) thiazol-5-yl] acetate 5g* **:** IR (KBr pellets) ν, cm^-1^: 3348 (N-H str), 3101 (Aromatic C-H str), 1714 (C=O str), 1610, 1529, 1487 (Aromatic C=C str), 707 (Aromatic Br str) .^1^H NMR (400MHz, CDCl_3_, δ ppm): 1.11, (t, 3H, CH_2_CH_3,_* J *:7.00 Hz,)_, _4.0, (s, 2H,CH_2_), 4.15, (q, 2H, CH_2_CH_3,_*J*: 7.1 Hz), 7.14 (t, 1H, Ar-Hd, *J*=4.7 Hz,), 7.33 (d, 1H,ArHc, *J*=3.6 Hz,), 7.49 (d, 2H, Ar-H_2’6’_*J*=8.86 Hz) 7.64, (d, 2H, Ar-H_3’5’_ , *J*=8.89 Hz ); 7.56(d, 1H, Ar-He, *J*=4.84 Hz) 10.35, (s, 1H, NH).^13^C NMR ( 400MHz, DMSO-d_6_) δ ppm (DEPT): 160.536 (C_2,_ Q), 112.535/112.317 (C_5_, Q/C_4’_, Q), 32.047(C_6,_ CH_2_), 169.825 ( C_7_, C=O), 60.905 (C_8_, CH_2_), 14.086 (C_9_, CH_3_), 140.325/141.282 (C_4_, Q/C_1’_, Q), 131.688 (C_2’ _and C_6’_, CH), 118.850 (C_3’­_and C_5’_, 2CH), 137.818 (Cb, CH), 127.841 (Cc, CH), 125.993 (Cd, CH), 124.855 (Ce, CH). ESI-MS (m/z):424/422 (M^+^+2/M^+^), 351/349 (100%, M^+^ - COOC_2_H_5_).

*Ethyl2-[2-(4-fluorophenylamino)-4-(thiophen-2-yl) thiazol-5-yl]acetate 5h :* IR(KBr pellets) ν, cm^-1^: 3166 (NH str), 2922 (C-H str) ; 1732 (C=O str), 1593, 1546, 1471 (C=N, Aromatic C=C str). ^1^H NMR (400MHz, CDCl_3_, δ ppm): 1.24 (t, 3H, CH_2_CH_3, _*J*: 7.13 Hz)_, _3.86, (s, 2H, CH_2_), 4.16-4.26 (q, 2H, CH_2_CH_3, _*J*: 7.11 Hz), 6.88-7.08 (q, 3H, Ar-H), 7.22-7.32, (m, 4H, Ar-H); 7.54-7.92(br, 1H, NH). ESI-MS (m/z): 362 (M^+^), 289 (100%, M^+^ - COOC_2_H_5_). Anal. Calcd%: (C_17_H_15_FN_2_O_2_S_2_): C, 56.34, H 4.17, N 7.73, S 17.69.Found (%): C 56.29, H 4.14, N 7.71, S17.72.

*Ethyl2-[2-(3-chloro-4-fluorophenylamino)-4-(thiophen-2-yl) thiazol-5-yl] acetate 5j :* IR(KBr pellets) ν, cm^-1^: 3332 (NH str), 2991 (C-H str), 1716 (C=O str), 1606, 1537, 1454 ( C=N, Aromatic C=C) str, 1211 (Ar-F str), 875 (Ar-Cl str). NMR (CDCl_3_, δ, ppm): 1.28 (t, 3H, CH_2_CH_3,_*J*: 7.14 Hz,)_, _3.89, (s, 2H,CH_2_), 4.19-4.24 (q,2H, CH_2_CH_3,_*J*: 7.01 Hz,), 7.04-7.10 (m, 2H, Ar-H), 7.17-7.22, (m, 1H, Ar-H); 7.32-7.34 (m, 2H, Ar-H), 7.48-7.51 (dd, 1H, Ar-H). MS, m/z: 397/395 (M^+^+2/M^+^), 324/322 (100%, M^+^ - COOC_2_H_5_). Anal. Calcd. for C_17_H_14_ClFN_2_O_2_S_2_, C, 51.45; H,3.56; N, 7.06; S, 16.16 found C, 51.48; H,3.49; N, 7.11; S, 16.11.

*Ethyl2-[2-(4-methoxy*p*henylamino)-4-(thiophen-2-yl) thiazol-5-yl] acetate 5k*: IR (KBr pellets) ν, cm^-1^: 3216 (NH str), 2892 (C-H str); 1721 (C=O, ester), 1589, 1566, 1482 (C = N, Ar C = C str). ^1^HNMR (400MHz, CDCl_3_, δ ppm): 1.19(t, 3H, CH_2_CH_3_,* J* = 7.02 Hz)_, _3.7 (s, 2H,CH_2_), 3.85 (s, 3H, OCH_3_) 4.2 (q, 2H, CH_2_CH_3,_*J *= 7.04 Hz), 6.9 -7.64 (m, 7H, Ar-H), 8.1 (br, 1H, NH).ESI-MS (m/z): 374 (M^+^), 301 (100%, M^+^ - COOC_2_H_5_)

*General synthesis of benzylidene thiosemicarbazones (8a-8g)*

The thiosemicarbazide 6 (0.01 mol) was dissolved in ethylene glycol (15 mL) in a MW test tube and substituted benzaldehydes **7** (0.01 mol) was added and the reaction mixture was exposed to microwaves at 100 °C (Power 100 W) for 30 sec. Thiosemicarbazones 8a-8g precipitated as a white solid was filtered and washed with alcohol and dried to obtain an analytically pure compound. The formation of these compounds was confirmed by their melting points, which were same as reported in the literature (31). 

*General synthesis of ethyl 2-[2-(2-(substituted benzylidene)hydrazinyl)-4-(thiophen-2-yl)thiazol-5-yl] acetates(9a-9g)*

The benzylidine thiosemicarbazone 8a-8g (0.01 mol) was dissolved in polyethylene glycol (10 mL) in a 100 mL conical flask and bromo ester 3 (0.01 mol) was added and placed in the microwave cavity and irradiated at 100 °C (Power 100 W) for 40 sec. The completion of the reaction was observed using TLC (n-hexane: ethyl acetate: 6: 4). 

The reaction was mixture cooled and mixed with sodium carbonate solution to obtain the pale yellow to yellow precipitate of thiazole acetates 9a-9g, which were filtered, washed with water, dried, and crystallized. Yield and melting points are reported in Table 1.

*Ethyl2-[-2-(2-(benzylidenehydrazinyl)-4-(thiophen-2-yl) thiazol-5-yl] acetate (9a):* IR(KBr pellets) ν, cm^-1^: 3217 (NH str), 3066 (Aromatic C-H str), 2981 (C-H str), 1722 (C=O str), 1595, 1573, 1487 (C=N, Aromatic C=C str). ^1^H NMR (400MHz, DMSO d_6_, δ ppm): 1.35 (t, 3H, CH_2_CH_3,_* J* :7.19 Hz,)_, _3.77, (s, 2H,CH_2_), 4.27 (q,2H, CH_2_CH_3,_*J*: 7.16 Hz,), 6.96 (s, 1H, N=CH), 7.18, (m, 1H, Ar-Hd);7.28-7.37 (m, 6H, 5Ar-H & 1Ar-He), 7.51 (m, 1H, Ar-Hc), 10.9 (s, 1H, NH). ESI-MS (m/z): 371 (M^+^), 268 (22%), 195 (100%). Anal. Calcd% (C_18_H_17_N_3_O_2_S_2_): C, 58.20; H, 4.61, N, 11.31; S, 17.26. Found (%): C, 58.15; H, 4.66, N, 11.27; S, 17.20.

*Ethyl2-[2-(2-(2-hydroxybenzylidene) hydrazinyl)-4-(thiophen-2-yl)thiazol-5-yl] acetate (9b) :* IR(KBr pellets) ν, cm^-1^: 3529 (OH str), 3178 (NH str), 3066 (Aromatic C-H str), 1732 (C=O str), 1591,1566, 1517, 1458 (C=N, Aromatic C=C str). ^1^H NMR (400MHZ, DMSO d_6_, δ ppm): 1.31 (t, 3H, CH_2_CH_3,_*J*: 7.16 Hz,)_, _3.71, (s, 2H,CH_2_), 4.24 (q,2H, CH_2_CH_3,_*J*: 7.15 Hz,), 6.45 (d, 1H, Ar-H_3_), 6.83 (t, 1H, Ar-H), 6.93 (d, 1H, Ar-H), 6.98 (s, 1H, N=CH), 7.1, (m, 1H, Ar-H), 7.23 (m, 1H, Ar-H), 7.3 (t, 1H, Ar-H), 7.45 (m, 1H, Ar-H),9.1-9.3 (br, 1H, OH) 10.3 (s, 1H, NH).ESI-MS (m/z): 387 (M^+^), 268 (9%), 195 (100%). 

*Ethyl2-[2-(2-(2-chlorobenzylidene) hydrazinyl)-4-(thiophen-2-yl)thiazol-5-yl] acetate 9c :* IR(KBr pellets) ν, cm^-1^: 3164 (NH str), 3062 (Aromatic C-H str), 2794 (C-H str), 1730 (C=O str), 1598 (C=N str), 1558, 1577, 1471(Aromatic C=C str), 852 (Aromatic C-Cl str). ^1^H NMR (400MHz, DMSO d_6_, δ ppm): 1.3 (t, 3H, CH_2_CH_3,_* J*: 7.14 Hz,)_, _3.67, (s, 2H,CH_2_), 4.25 (q,2H, CH_2_CH_3,_*J*: 7.01 Hz,), 6.63 (dd, 1H, Ar-H), 6.8 (t, 1H, Ar-H), 6.93 (d, 1H, Ar-H), 7.10 (s, 1H, N=CH), 7.12, (m, 1H, Ar-H);7.21 (m, 1H, Ar-H), 7.32 (d, 1H, Ar-H), 7.4 (m, 1H, Ar-H), 10.1 (s, 1H, NH).ESI-MS m/z: 406/404 (M^+^+2, M^+^), 268 (11%), 195 (100%). 

*Ethyl 2-[2-(2-(4-dimethylaminobenzylidene) hydrazinyl)-4-(thiophen-2-yl)thiazol-5-yl] acetate (9e);* IR (KBr pellets) ν, cm^-1^: 3184 (NH str), 3082 (Aromatic C-H str), 2977 (C-H str), 1735 (C=O str), 1600 (C=N str), 1583, 1527, 1485 (Aromatic C=C str). ^1^H NMR (400 MHz, DMSO d_6_, δ ppm): 1.34 (t, 3H, CH_2_CH_3,_*J *:7.04 Hz,), 2.99 (s, 6H, N(CH_3_)_2_), 3.77, (s, 2H,CH_2_), 4.22(q,2H, CH_2_CH_3,_*J*: 7.08 Hz,), 6.63 (dd, 2H, Ar-H_2’_& H_6’_, *J *= 8.36Hz), 7.01 (s, 1H, N=CH),7.1 (m, 1H, Ar-H), 7.26 (d, 2H, Ar-H_3’_& H_5’_, *J*=8.36Hz), 7.34, (d, 1H, Ar-H), 7.44 (m, 1H, Ar-H), 11.2 (s, 1H, NH).MS m/z: 414 (M^+^),268 (14%), 195 (100%). Anal. Calcd% (C_20_H_22_N_4_O_2_S_2_): C, 57.95; H, 5.35; N, 13.52; S, 15.47. Found (%): C, 58.01; H, 5.31; N, 13.57; S, 15.52.

*Ethyl2-[2-(2-(4-methoxybenzylidene) hydrazinyl)-4-(thiophen-2-yl) thiazol-5-yl] acetate (9f) :* IR(KBr pellets) ν, cm^-1^: 3300-2700 (OH, str), 3147 (NH str), 3047 (Aromatic C-H str), 2788 (CH_2 _str), 1735 (C=O str), 1610 (C=N str), 1566, 1481 (Aromatic C=C str). ^1^H NMR (400MHz, DMSO d_6_, δ ppm): 1.3 (t, 3H, CH_2_CH_3,_* J* :7.14 Hz), 3.81 (s, 1H, ArOCH_3_) 3.86, (s, 2H, CH_2_), 4.23 (q,2H, CH_2_CH_3,_*J*: 7.16Hz,), 6.85 (d, 1H, Ar-H_3 & _H_5_, *J*: 8.73 Hz), 6.85 (t, 1H, Ar-H), 7.07 (s, 1H, N=CH), 7.09 (s, 1H, Ar-H_d_), 7.30, (m, 1H, Ar-H_e_), 7.33 (d, 2H, Ar-H_2’_&H_6’_), 7.4 (4, 1H, Ar-H), 10.3 (s, 1H, NH).ESI-MS (m/z): 401 (M^+, ^18%), 268 (20%), 195 (100%). 

*Pharmacological activity*

*Anti-inflammatory activity:* Anti inflammatory activity was determined for all synthesized compounds using the carrageenan induced rat hind paw edema method (32). Wister rats (160-230 g) were divided into different groups: six for each group for control, standard, and test compounds. The suspensions of test compounds were prepared in Tween 80 (10%v/v). Next, 0.1 mL of freshly prepared 1 mg/mL carrageenan (an irritant) was injected into sub-planter tissue of the hind paw of the Wister rats of all groups to produce edema. Standard drug indomethacin and the test compounds (100 mg/kg body weight) were administered orally to different groups immediately after injecting the carrageenan, while the control group received the same volume of Tween 80 solution. The paw volume was recorded immediately after the oral administration of the compounds and 3 h after the oral administration. The anti-inflammatory activity of the compounds was calculated as the percentage inhibition of edema using the equation: ((Vc-Vt)/Vc)*100, where Vc is the increase in the paw volume of the control and Vt is the increase in the paw volume after administration of the compounds. 

*Analgesic activity:* Analgesic activity was performed using the acetic acid-induced writhing assay (33). Standard drug aspirin, test samples at 100 mg/kg body weight, and the vehicle (Tween 80, 10% v/v) used for the preparation of samples were administered to a different group of Swiss albino mice (six in each group). After 30 min, 0.5% acetic acid was injected intraperitonially at a dose of 0.1 mL/10g body weight to induce writhing, and the writhing episodes were recorded for 20 min. The percentage protection against the writhing episodes in the standard and drug-treated animals were recorded and calculated using the formula: % Inhibition = (1- W_t_/W_c_) x 100, where Wt and Wc are the means of the writhing episodes in the test and control groups, respectively.

*Antioxidant activity:* Some of the NSAIDs showed anti-inflammatory activity by reduction of super oxide radicals. Hence, antioxidant activity was determined for the newly synthesized compounds by reduction of diphenyl-2-picrylhydrazyl (DPPH) in methanol (516 nm) (34). Assays were carried out by mixing a solution of 2.0 mL of 100 μm DPPH in methanol, 1 mL of methanol (control), or test compounds. The mixture was incubated at room temperature for 20 min, and then the absorbance was recorded at 516 nm. The assay was repeated three times. Ascorbic acid was used as the standard control. Antioxidant activity was calculated as the percent inhibition of DPPH using the following formula: inhibition (%) = ((Ac-As)/Ac) 100, where Ac represents the absorbance of the control and As represents the absorbance of the sample. IC 50 valve is the concentration of test samples required to scavenge 50% of the radicals. The dosage of extract is expressed in μg/mL for the assay mixture. 

*Molecular modeling study*

Synthesized molecules were subjected to molecular modeling studies using the Molecular Operating Environment (MOE) 2013.08 (35) (MOE 2014) software package; the license was purchased from Chemical Computing Group Inc, Montreal, QC, Canada. The Leadit 2.1.2 software license was purchased from BioSolveIT GmbH, Germany. (36).

*Molecular docking studies with Leadit 2.1.2*

All compounds were built and saved as Mol2. The crystal structure of the COX-1 and COX-2 enzymes complexed with indomethacin was downloaded from a protein databank (pdb code = 4COX). The protein was loaded into Leadit 2.1.2 and the receptor components were chosen by the selection of chain A as a main chain that is complexed with indomethacin. The binding site was defined by choosing indomethacin as a reference ligand to which all coordinates were computed. Amino acids within radius 6.5 A were selected in the binding site. All chemical ambiguities of residues were left as default. Ligand binding was driven by enthalpy (classic triangle matching). For scoring, all default settings were restored. Intra-ligand clashes were computed by using clash factor = 0.6; maximum number of solutions per iteration = 200; and maximum of solution per fragmentation = 200. The base placement method was used as a docking strategy. 

*Molecular docking studies with MOE 2013.08*

All compounds were built and saved as MOE. A rigid receptor was used as the docking protocol, and both receptor–solvents were kept as a “receptor”. A triangle matcher was used as a placement method. Two rescorings were computed: rescoring 1 was selected as London dG, while rescoring 2 was selected as affinity. Force field was used as a refinement. The molecular surfaces were computed to determine the lipophilicity near the fixed best conformations for the docked compounds and were created within 4.5 A. The cutoff was 2.5, and speed was set as the default.

## Results and Discussion

*Chemistry*

The general procedure employed in the preparation of the title compounds 5a-5k and 9a**-**9g is outlined as Scheme 1 and 2, respectively. The key intermediate ethyl 3-bromo-3-(2-theinoyl) propionate 3 was prepared starting from thiophene by succinoylation, esterification, and bromination. Substituted phenylthioureas 4 were synthesized by the reaction of appropriate substituted aniline with benzoyl chloride and ammonium thiocynate in acetone. Araldehydethiosemicarbazones 8a-8g were prepared by the condensation of related aldehyde with thiosemicarbazide in ethylene glycol under microwave irradiation using a few drops of concentrated sulfuric acid. Substituted phenylthioureas and araldehydethiosemicarbazones were then treated separately with bromo ester 3 in PEG-400 under microwave irradiation to yield Ethyl-2-[2-substituted phenylamino 4-(thiophen-2-yl) thiazol-5-yl] acetate 5a-5k and ethyl 2-[2-(arylhydrazino)-4-(thiophen-2-yl) thiazol-5-yl] acetates 9a-9g, respectively (Scheme 1 and 2).

The physical constants of newly synthesized substituted thiazoles are tabulated in Table1. The structure of substituted thiazoles was confirmed by IR, NMR (^1^H and ^13^C), and mass spectra of the compounds. IR spectra of compound 5g showed strong stretching bands at 3294, 1730, 1594, and 1068 cm^-1^ for the NH, COOEt, C=N, and C-S groups, respectively. The lack of the absorption band matching to a carbonyl stretching frequency of the parent ethyl 3-bromo-3-(2-theinoyl) propionate and NH_2_ stretching frequencies of phenyl thiourea evidently established the formation of 2-substituted aminothiazole acetates. The proton NMR spectra of compound 5g showed a singlet at δ 10.35 for the proton of the NH group. The aromatic protons of 4-(thiophen-2-yl) ring emerged as two doublets at δ 7.33 (*J* = 3.6 Hz) and 7.56 (*J* = 4.84 Hz) and one triplet at δ 7.14 (*J* = 4.7 Hz) integrating for one proton respectively, and as a two doublets at δ 7.49 (*J* = 8.86 Hz) and δ 7.64 (*J* = 8.89 Hz) integrating for four protons of the 4-bromophenyl ring. The C=NH proton of compounds 9a-9g emerged as a singlet at δ 6.96-7.1. The NH resonated as a singlet in the range of δ 10.1-11.2. IR and ^1^H-NMR of all the other compounds proved the structure of all the other compounds, and their spectral data are depicted in the experiment section. 13C NMR and distortionless enhancement by polarization transfer (DEPT) spectra of compounds 5a, 5b**, **and 5g also supported the structure assigned to the compounds. The mass spectra were recorded for all the compounds and showed strong molecular ion peaks; they were similar to their respective molecular mass. The common fragmentation of the compounds was observed by elimination of an ethoxycarbonyl group at C5 acetate to form fragment A, which is a base peak in all the mass spectra, indicating its stability due to its rearrangement to form a thiazine nucleus (B). Subsequent cleavage of S-C2 and C2- N leads to four membered cyclic fragment ions (C) (Scheme 3). Benzylidinehydrazinothiazolyl acetates 9 also showed the same fragmentation after the elimination of the -ArCN group (Scheme 4) to yield fragment F from fragment E.

*Pharmacological activity*

Title compounds were screened for anti-inflammatory activity by a carrageenan-induced rat paw edema process. The anti-inflammatory activity of tested compounds (Table 2) demonstrated variable activity and, interestingly, most of the 2-arylamino thiazole acetates showed significant anti-inflammatory activity, whereas the aryledine compounds were less active. Compounds with a 4-flurophenyl derivative **(**5h) were found to be most active with 78.8% inhibition. Substitution of the flouro group at the para position with the bromo group (5g) and chloro group (5c) slightly decreased the activity. However, the compounds with halogens at the para positions of the 2-arylamino group (5c, 5g, 5h, 5i, and 5j) showed good anti-inflammatory activity due to enhanced lipophilicity, as evident from the docking studies and their binding mode analysis. Further, the substitution of electron donating groups **(**5f and 5k) at the para position decreased activity substantially. All the ortho substituted compounds **(**5a and 5f) exhibited poor anti-inflammatory effects, probably due to steric hindrance. Ethyl 2-[2-(substituted benzylidenehydrazino)-4-(thiophen-2-yl) thiazol-5-yl] acetates **(**9a-9g) showed moderate to mild anti-inflammatory activity.

Newly synthesized thiazole acetates were also screened for analgesic activity by writhing method. From the results depicted in Table 2, it was observed that a change in position and type of substitution at the 2-aryamino group influenced activity. The halogen substituted at the fourth position of phenylamino derivatives (5a, 5g, and 5h) showed good analgesic activity comparable to aspirin. The 4-methyl (5d) and 4-methoxyphenyl **(**5k) derivatives showed reduced anti-inflammatory activity. Other compounds (5j and 5b) were found to have relatively good analgesic activity. Compounds **(**9a -9g) showed weak analgesic activity.

It is well established that free radicals play a vital role in the inflammatory process. Reactive oxygen species, superoxide anions, hydrogen peroxide, and hydroxyl radicals are produced as byproducts of a variety of aerobic metabolism pathways that are considered to be involved in inflammation (37). Many studies have suggested that agents with the ability to protect against these free radicals may be useful to protect against inflammation (38). Hence, compounds were also screened for antioxidant activity by the DPPH method. The highest radical scavenging activity was shown by 9a with IC 50 = 8.25 μg/mL. Any substitution on the phenyl ring at the second position of the thiazole ring decreased the activity. The benzylidinehydrazino substituted by compounds (9a-9g) showed better radical scavenging activity than aryl amino substituted compounds (**5a-**5k), indicating that the anti-inflammatory activity by compounds 5a-5k is not a result of free radical inhibition mechanisms. 

*Molecular docking results*

All of the synthesized compounds (5a-5k) and (9a-9h) were evaluated to identify their hypothetical binding mode on the X-ray crystal structure of the COX-2 enzyme and compared to the docking results of indomethacin. From the docking results (Table 3), it can be seen that the five top-ranked compounds with the highest docking score are those with the highest anti-inflammatory effect as well (5c, 5g, 5h, 5i, 5j). Moreover, they showed a common binding mode in which the electronegative sulfur atom in the thiophene ring participated with an electrostatic interaction with the positively charged guanidine moiety of the Arg 120 residue. In addition, the same sulfur atom had a hydrogen bond with a hydrogen atom from the hydroxyl group of Tyr 355. This is different from indomethacin, which showed two types of interactions in the same position: the first one was an electrostatic interaction with Arg 120, and the second one was a hydrogen bond with Tyr 355. Indomethacin had the strongest docking score compared to all synthesized compounds (–27.25 kcal/mol score). The lipo score was more than that of indomethacin and the clash penalty score was close to compound 5i. This means that the same binding mode of indomethacin was achieved by the active compounds as well (Figure 1). The 2-arylamino–NH showed a hydrogen bond with the oxygen atom of Ser 530 in compounds 5g, 5h, and 5j. The computed docking scores were correlated with anti-inflammatory activities. For example; compound 5g with the highest anti-inflammatory activity also had the highest score (–21.31 kcal/mol). 

Clash penalty is a parameter that measures the extent of clashes formed by the docked compounds. The lower the clash score, the better the fit in the active site. Ibuprofen has a very low clash score (= 5.78). The top five compounds according to the clash score in ascending order are: compound 5g = 5.01; compound 5h = 5.21; compound 5c = 6.49; compound 5j = 6.67; and compound 5i = 7.21, respectively. To compare selectivity towards COX-1 and COX-2, the top five active compounds (5c, 5g, 5h, 5i, 5j) were also subjected to docking studies against COX-1 (Figure 2). Indomethacin showed a higher docking score (–22.39 kcal/mol) in comparison to the test compounds (Table 4). Although indomethacin did not show the highest lipophilic contribution, it had a lower clash score (7.22). The strongest interactions with the COX-1 binding site was observed with compound 5g, compound 5h, and compound 5i due to the formation of electrostatic interaction with the guanidine moiety of Arg 120, which supported their fitting in addition to the hydrogen bond forming with Ser 530. However, the comparison of the docking and clash scores of indomethacin and the tested compounds (5c, 5g, 5h, 5i, 5j) on COX-1 and COX-2 revealed that the title compounds have better COX-2 selectivity than COX-1

The other group of compounds (9a-9h) showed very high clash penalty score ranging from 12.48 for compound 9f to 14.60 for compound 9h. Both high clash scores and the lowest docking scores for compounds 9a-9h were not promising results.

Another feature was observed in the top five active anti-inflammatory compounds: all of them have a para-substituted halide atom in the phenyl moiety. In order to find a good explanation for the molecular docking of all compounds into COX-2, a docking study was carried out using the MOE 2013.08 software. The best conformation for each one of the five compounds was kept inside the active site, and they were found to be aligned well. Upon computing the lipophilicity maps of the binding site, it was found that all the phenyl moieties with the para-halogen atoms were oriented toward the most lipophilic part of the active site, which means that the activity of these compounds with para-halogen atoms may refer to their lipophilic contribution in the active site (Figure 3). Moreover, this was also confirmed by computing the lipophilic contribution of the docked compounds using Leadit 2.1.2. The lipo score was an indication of this feature: the top five compounds had the highest lipo scores (Table 3), which were more than that for indomethacin. The other set of compounds, such as 5a, 5b, 5d, 5e, 5f, and 5k, shared another binding mode in which the acetate moiety played a key role. 

Finally, compounds 9a-9h showed different hydrogen bond formations with the accepting C=N group of the benzylidene group. This enabled them to form a hydrogen bond with both Tyr 355 and Arg 120, but the large distances indicated weak bonding. 

## Conclusions

In the present study, new ethyl 2-[2-substituted-4-(thiophen-2-yl)thiazol-5-yl] acetates were synthesized with good yields using microwave assisted synthesis in a short time and they were evaluated for their anti-inflammatory, analgesic, and antioxidant activities. In general, ethyl 2-[2-substituted phenylamino-4-(thiophen-2-yl)thiazol-5-yl] acetate** (**5a-5k**)** showed good anti-inflammatory activity and, more specifically, compounds with para halogen substitutions (5c, 5g, 5h, 5i, and 5j) emerged as the most active compounds among all compounds screened. Further, in silico docking studies were performed using Leadit 2.1.2 software to identify the hypothetical binding pattern of the synthesized compounds with the COX-2 enzyme. Anti-inflammatory activity of the screened compounds paralleled the molecular docking results. All the active compounds illustrated strong hydrogen bonding between the sulfur of thiophene and Tyr 355 and Arg 120 of COX-2, in addition to the electrostatic interaction between the electronegative sulfur atom of thiophene and the positively-charged guanidine of Arg 120. Additionally, docking studies with MOE 2013.08 software showed good alignment of these compounds in the active site of COX-2.
